# Ultrasound examination of asymmetry in temporomandibular disorders

**DOI:** 10.1016/j.clinsp.2026.100963

**Published:** 2026-04-25

**Authors:** Szilvia Mihályi, Flóra Borbála Horváth, Ákos Wágner, Judit Anna Kolonics, László Simonffy, Péter Hermann

**Affiliations:** a Semmelweis University, Doctoral College, Budapest, Hungary; b Dentideal Ltd., Dentideal Oral Surgery and Dental Clinics, Budapest, Hungary; c Semmelweis University, Faculty of Dentistry, Educational Center, Dentoalveolar Surgery Department, Budapest, Hungary; d Semmelweis University, Faculty of Dentistry, Clinic for Prosthodontics, Budapest, Hungary

**Keywords:** Ultrasonography, Asymmetry, Temporomandibular disorders, Prevention, Masticatory muscles

## Abstract

**Purpose:**

Ultrasonography is a non-invasive, radiation-free, and cost-effective imaging modality for evaluating the Temporomandibular Joint (TMJ) and Masticatory Muscles (MM). Despite its clinical potential, a standardized quantitative ultrasound protocol for assessing masticatory system asymmetry in Temporomandibular Disorders (TMD) is lacking. This pilot study aimed to evaluate the feasibility of a standardized ultrasound protocol for the quantitative assessment of masticatory system asymmetry and to explore its preliminary associations with TMD-related symptoms.

**Materials and methods:**

Fifteen young adult participants (aged 19–26 years) underwent bilateral ultrasound examination of the masticatory muscles (masseter, temporalis, lateral pterygoid, and sternocleidomastoid muscles) and TMJ structures, including interarticular spaces and articular components. Measurements were performed by trained examiners using standardized anatomical landmarks. Asymmetry was quantified using percentage difference indices and aggregated into a composite anatomical asymmetry score. Proposed asymmetry reference values were applied for descriptive purposes only, and all statistical analyses were conducted on an exploratory, hypothesis-generating basis.

**Results:**

The composite anatomical asymmetry index differed from symmetry (mean ± SD: 18.45% ± 17.13%). An association was observed between anatomical asymmetry and joint sounds (crepitation) (Spearman’s ρ = 0.88), while pain showed a lower-magnitude association (ρ = 0.51).

**Conclusion:**

This pilot study demonstrates the feasibility of a standardized ultrasound-based protocol for the quantitative assessment of masticatory system asymmetry. The findings are exploratory and intended to support methodological development and hypothesis generation. Further validation in larger, blinded study populations is warranted.

## Highlights

• Standardized ultrasound protocol for TMD asymmetry assessment.
• Quantitative indices for muscle and TMJ asymmetry analysis.
• Ultrasound asymmetry linked to TMD symptoms (exploratory).
• Feasibility of multimodal ultrasound in the masticatory system study.

## Introduction

### Definition

The existing literature differentiates between Temporomandibular Disorders (TMD) of articular origin and those primarily involving the masticatory muscles. However, the Temporomandibular Joint (TMJ) and the associated musculature function as a closely integrated unit, commonly referred to as the masticatory system. The TMJ, as one of the most frequently used joints in the human body, is highly dependent on coordinated muscular activity.

The masticatory system is involved in a wide range of essential functions, including mastication, swallowing, mouth opening and closing, protrusive and retrusive movements, lateral mandibular movements, phonation, facial expression, breathing, and maintenance of middle ear pressure.

### Etiology

Structural or functional alterations affecting any component of the masticatory system may contribute to the development of temporomandibular disorders. Prolonged exposure to such alterations may be associated not only with local symptoms but also with psychosocial factors, including anxiety, depression, and stress, which have been reported to show bidirectional associations with TMD.^1^, ^2^, ^3^

Masticatory muscle thickness represents an important etiological factor in the development and manifestation of TMD. Variations in muscle thickness ‒ whether hypertrophy, atrophy, or asymmetry ‒ may alter local biomechanical conditions within the TMJ and contribute to pathological changes.

Muscle hypertrophy, often associated with parafunctional behaviors such as bruxism or unilateral chewing, may increase occlusal forces and lead to excessive joint loading.^4^ These changes have been linked to articular disc displacement, condylar remodeling, and capsular inflammation. Conversely, muscle atrophy has been observed in disuse, pain-induced hypofunction, or iatrogenic variables like the use of botulinum toxin. Reduced muscle thickness may impair stabilization of the masticatory system and result in joint instability or altered mandibular kinematics.^5^

Asymmetry of the TMJ and masticatory system may arise from congenital or developmental anomalies, trauma, functional overload (e.g., bruxism or unilateral chewing), inflammatory or degenerative pathology, treatment-related factors, or postural and neurological influences.^6^, ^7^, ^8^, ^9^ Unequal biting forces may subsequently lead to mandibular deviation and unilateral condylar overload. Imaging studies have demonstrated associations between masseter muscle thickness and condylar position, suggesting that muscle morphology may directly influence joint position and stability.^10^

### Ultrasonography in general

Ultrasonography is a non-invasive, radiation-free, and cost-effective imaging modality for the evaluation of the temporomandibular joint and the masticatory system. In contrast to computed tomography and magnetic resonance imaging, ultrasonography does not involve ionizing radiation and can therefore be repeated, when necessary, particularly in preventive settings, early disease stages, and follow-up examinations.

In addition, ultrasonography allows dynamic, real-time assessment of both joint and muscle function, enabling visualization of mandibular movement, muscle contraction, and functional alterations during examination. Therefore, as previously reported, ultrasonography may be incorporated into routine dental examinations and used as a chairside imaging tool for functional assessment of the temporomandibular joint and masticatory muscles.^11^

### Ultrasonography of the masticatory system

Masticatory muscles that can be examined using ultrasonography include the masseter, temporalis, lateral pterygoid, and sternocleidomastoid muscles.^12^, ^13^, ^14^ Quantitative ultrasonographic assessment of muscle thickness, width, stiffness, and functional behavior provides valuable information on oral motor function and craniomandibular dynamics. These parameters may vary considerably among individuals with temporomandibular disorders, particularly in the presence of myofascial pain or parafunctional activity. Several studies have reported reduced maximal mouth opening, thinner sternocleidomastoid and masseter muscles, as well as increased muscle tone or stiffness in patients with TMD.^5^^,^^15^, ^16^, ^17^

Such findings suggest that ultrasonographic evaluation of masticatory muscles may reflect functional imbalance as well as adaptive or pathological changes within the masticatory system. Ultrasonography is also used to assess the TMJ, with one of the most evaluated parameters being the interarticular joint space, often described as the capsule-condyle distance, which may reflect inflammatory changes or altered joint mechanics. Bilateral comparison of these measurements may assist in the early identification of anatomical alterations.^18^^,^^19^

Beyond static assessment, ultrasonography allows dynamic, real-time visualization of muscle contraction and joint movement, which is not feasible with conventional radiographic methods. This capability supports its use in functional evaluation and longitudinal monitoring of the masticatory system. Differences in masticatory muscle thickness have been demonstrated between temporomandibular disorders patients with and without bruxism, emphasizing the relevance of quantitative muscle assessment in specific clinical subgroups.^22^ Furthermore, dynamic quantitative imaging approaches have been proposed to investigate whether ultrasonography-based muscle measurements could serve as objective biomarkers in TMD-related conditions.^23^ Ultrasonography has also been applied to monitor treatment-related changes in masticatory muscle morphology, with measurable alterations in masseter muscle thickness reported following botulinum toxin A treatment in patients with bruxism.^24^

Taken together, these findings indicate that ultrasonography provides a versatile, non-invasive, and radiation-free method for evaluating both structural and functional aspects of the masticatory system. However, despite its increasing application, standardized and clinically applicable protocols for the comprehensive quantitative assessment of bilateral asymmetry across masticatory muscles and TMJ structures remain limited.

### Knowledge gap

Despite increasing use of ultrasonography, standardized and clinically applicable protocols for the quantitative evaluation of masticatory system asymmetry remain limited.^23^

### Aim

Prevention and early diagnosis represent important objectives in contemporary TMD research. Approximately 60%–70% of the general population exhibits signs of temporomandibular disorders. However, only about 25% of affected individuals report or are aware of symptoms.

It is particularly noteworthy that a significant proportion of patients affected by TMD remain asymptomatic during the early stages of the condition.^20^ Early diagnosis and timely treatment of TMD are essential so that invasive procedures can be avoided. This is particularly important because untreated patients often do not show spontaneous improvement in their symptoms over time.^21^

Accordingly, the aim of this pilot study was to evaluate the feasibility of a standardized ultrasound-based protocol for the quantitative assessment of masticatory system asymmetry and to explore its preliminary associations with temporomandibular disorder-related clinical symptoms. Rather than establishing diagnostic criteria, the present investigation focuses on methodological development and hypothesis generation to support future larger-scale studies.

## Materials and methods

### Study design and participants

In accordance with a standardized ultrasound examination protocol, this pilot feasibility study included 15 young adult participants. The study population consisted of 11 females and 4 males between the ages of 19 and 26 years. Participants were recruited on a voluntary basis through a university-affiliated platform using institutional communication channels. No personal data was accessed prior to voluntary participation, and recruitment procedures complied with applicable data protection and privacy regulations.

Ultrasound examinations were conducted at the Dentideal Oral Surgery and Dental Clinic. The study was approved by the Hungarian Ethics Committee of the Medical Research Council. This pilot observational study was reported in accordance with the STROBE (Strengthening the Reporting of Observational Studies in Epidemiology) guidelines.

All participants completed a written informed consent form, a structured medical history form, and a standardized questionnaire assessing Temporomandibular Disorder (TMD) ‒ related symptoms prior to the ultrasound examination. Prior to data acquisition, a preliminary ultrasound scan was performed for each participant to familiarize the examiner with individual anatomical variations and to optimize transducer positioning and image quality. Data obtained during this preliminary scan were not included in the outcome assessment.

### Clinical symptom assessment

Clinical symptoms related to temporomandibular disorders were assessed using the standardized questionnaire. Pain was defined as the presence of spontaneous or movement-related pain in the temporomandibular joint or masticatory muscles. Joint sounds referred to patient-reported or clinically perceived clicking or crepitus during mandibular movement. Limited mouth opening was defined as a subjective sensation of restricted mandibular opening reported by the participant. Additional symptoms were recorded, including mandibular deviation during opening, defined as a visible deviation of the mandible from the midline, and tinnitus, defined as a subjective perception of ear noise not attributable to an acute otologic condition. All clinical symptoms were documented dichotomously as present or absent.

For descriptive purposes, participants were categorized during data evaluation into the following groups: (1) Participants with asymmetry and clinical symptoms, (2) Participants with asymmetry but without reported symptoms, and (3) Participants without reported symptoms or detectable asymmetry. This categorization was applied after data acquisition and was not used to guide measurements or influence data collection.

### Ultrasonographic examination

The study included ultrasonographic examination of the masticatory muscles (masseter, temporalis, lateral pterygoid, and sternocleidomastoid muscles), assessment of interarticular spaces (anterior capsule-condyle and lateral capsule-condyle distances), and evaluation of Temporomandibular Joint (TMJ) anatomical features on both sides. Participants were seated upright in the dental chair to replicate physiological conditions. Ultrasound examinations were performed using a GE Venue Go system equipped with a high-frequency linear transducer (GE L4-12T-RS, 3.4–12.6 MHz) in musculoskeletal mode, with an imaging depth of 4–5 cm. This setting allowed visualization of both superficial and deep muscle layers as well as joint structures.

The transducer was positioned perpendicular to the skin, and care was taken to avoid excessive pressure during measurements. Adequate ultrasound gel was applied to optimize image quality. Variations in probe pressure, angulation, or movement were minimized, as these factors may reduce image clarity, impair visualization of fascial boundaries, and decrease measurement reliability.

### Masticatory muscles

Muscle Thickness (MT) and Muscle Width (MW) were measured bilaterally during relaxation, maximal closing, and maximal mouth opening, with the exception of the lateral pterygoid muscle, which was evaluated only in the open-mouth position due to anatomical accessibility.

The muscle thickness was measured as the distance (in mm) between the superficial fascia and the deep fascia, as standardized landmarks.

#### Masseter muscle

Masseter muscle thickness and width were measured at the upper, middle, and lower portions using a linear probe during rest, mouth opening, and clenching. Measurements were obtained as the maximal distance between the outer and inner fasciae, which appeared as echogenic lines framing the muscle tissue.

##### Upper part of masseter

**Fig. 1:**
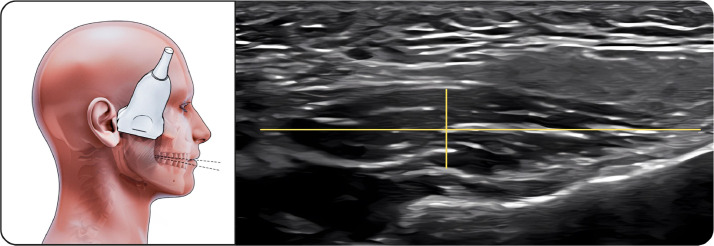
Upper part of masseter muscle. The figure on the left illustrates the upper portion of the masseter muscle in the resting position during ultrasonographic examination, while the image on the right shows the corresponding ultrasound image with standardized measurements. The linear transducer is positioned along the zygomatic arch and slightly moved caudally to visualize the muscle belly. On the ultrasound image, the parotid gland is visible superficial to the masseter muscle, serving as an anatomical reference structure. Muscle thickness and width are measured as the distance between the superficial and deep fasciae, which appear as echogenic boundaries surrounding the hypoechoic muscle tissue.

The transducer was placed along the zygomatic arch and moved slightly caudally (Fig. 1). The hypoechoic muscle with clearly defined superficial fascia and bony reflection was visualized, showing minimal thickness change during contraction.

##### Middle part of masseter

**Fig. 2:**
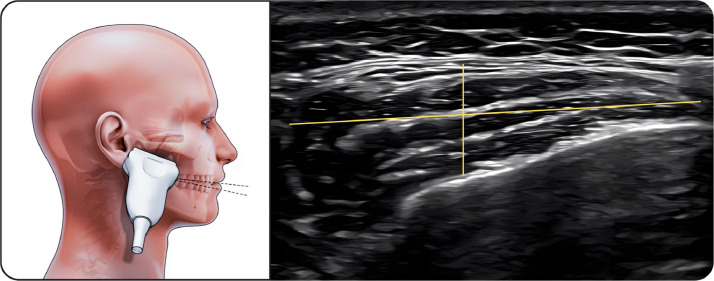
Middle part of masseter muscle. The figure on the left illustrates the middle portion of the masseter muscle in the resting position during ultrasonographic examination, while the image on the right shows the corresponding ultrasound image. The linear transducer is positioned along the zygomatic arch at the level of the condylar notch and adjusted to the muscle belly. The masseter muscle is visualized as a hypoechoic structure bordered by clearly identifiable superficial and deep fasciae. This region represents the thickest portion of the masseter muscle and was used for standardized bilateral thickness and width measurements.

The transducer was positioned along the zygomatic arch to identify the condylar notch and then adjusted to the muscle belly (Fig. 2). This region demonstrated the greatest thickness increase during clenching.

##### Lower part of masseter

**Fig. 3:**
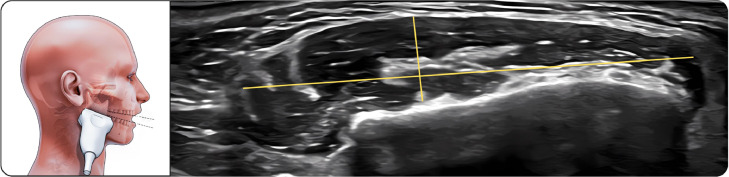
Lower part of masseter. The figure on the left illustrates the lower portion of the masseter muscle in the open-mouth position during ultrasonographic examination, while the image on the right shows the corresponding ultrasound image. The linear transducer is aligned with the long axis of the mandibular body near the mandibular angle to visualize the inferior part of the muscle. The masseter muscle appears as a hypoechoic structure with clearly identifiable superficial and deep fascial boundaries. Measurements of muscle thickness and width were obtained in this position using standardized anatomical landmarks.

The transducer was aligned with the long axis of the mandibular body near the angle of the mandible (Fig. 3). The muscle appeared slightly thinner than the middle portion but remained well visualized with distinct fascial borders.

#### Temporalis muscle

**Fig. 4:**
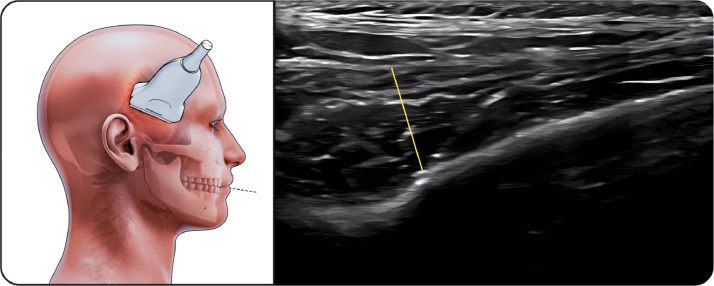
Temporalis muscle. The figure on the left illustrates the temporalis muscle during ultrasonographic examination during maximal intercupation, while the image on the right shows the corresponding ultrasound image. The linear transducer is positioned along the upper border of the zygomatic arch and moved cranially to visualize the temporalis muscle belly. The temporalis muscle appears as a hypoechoic structure containing echogenic connective tissue septa, which are visible within the muscle. Muscle thickness measurements were obtained using standardized anatomical landmarks under resting conditions.

The linear probe was positioned along the upper border of the zygomatic arch and moved cranially (Fig. 4). At rest, the temporalis muscle exhibited homogeneous echotexture with clearly defined fascial margins. During maximal intercuspation, a mild increase in muscle thickness was observed, while mandibular opening resulted in elongation and reduced thickness, consistent with muscle relaxation.

#### Sternocleidomastoid muscle

**Fig. 5:**
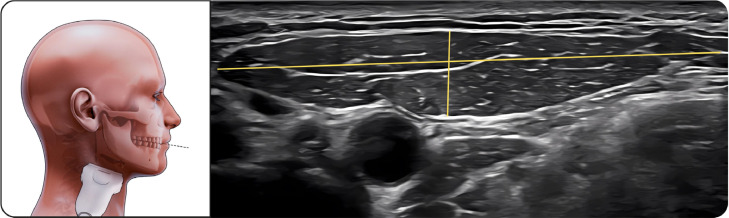
Sternocleidomastoid muscle. The figure on the left illustrates the sternocleidomastoid muscle during ultrasonographic examination in the closed-mouth position, while the image on the right shows the corresponding ultrasound image. The linear transducer is positioned on the lateral aspect of the neck, approximately midway between the mastoid process and the clavicle. On the ultrasound image, the sternocleidomastoid muscle appears as a well-defined hypoechoic structure. The carotid artery is visible medial to the muscle as a round, anechoic structure and serves as an important anatomical landmark during examination. Muscle thickness measurements were obtained bilaterally using standardized anatomical landmarks.

The linear transducer was placed on the lateral neck approximately halfway between the mastoid process and the clavicle, about 5 cm from the trachea and 3 cm from the mandible (Fig. 5). At rest, the sternocleidomastoid muscle appeared thin and well defined, thickening during contraction and elongating during stretching.

#### Lateral pterygoid muscle

**Fig. 6:**
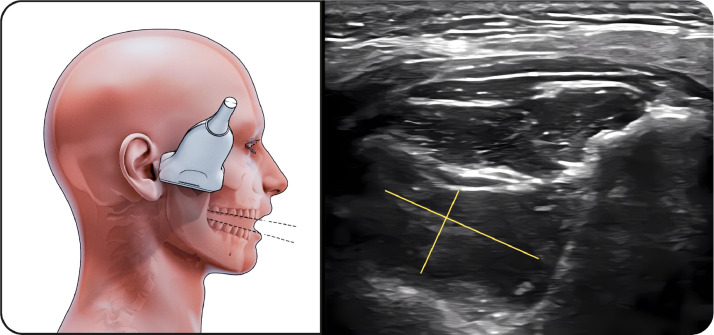
Lateral pterygoid muscle. The figure on the left illustrates the lateral pterygoid muscle in the open-mouth position during ultrasonographic examination, while the image on the right shows the corresponding ultrasound image. During mandibular opening, anterior condylar translation facilitates sonographic visualization of the lateral pterygoid muscle. The lateral pterygoid muscle appears as a triangular hypoechoic structure adjacent to the lateral pterygoid plate. A more superficial soft tissue layer, corresponding to the deep portion of the masseter muscle and adjacent connective tissue structures, is visible above the lateral pterygoid muscle and serves as an anatomical reference during examination. Measurements were obtained exclusively in the open-mouth position due to anatomical accessibility.

The lateral pterygoid muscle was best visualized in the open-mouth position. Mandibular opening allowed anterior translation of the condyle, creating an acoustic window for sonographic assessment. The probe was placed along the zygomatic arch and moved caudally to visualize the coronoid and condylar processes. The lateral pterygoid muscle appeared as a triangular structure connected to the lateral pterygoid plate (Fig. 6).

#### Interarticular space

**Fig. 7:**
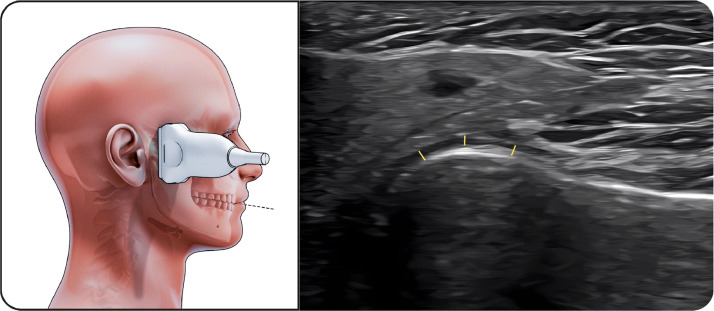
Lateral capsule-condyle distances. The lateral capsule-condyle distance is defined as the shortest linear distance between the hyperechoic joint capsule and the cortical surface of the mandibular condyle, assessed using a longitudinal ultrasonographic scan. On the ultrasound image, the mandibular condyle is identified by its hyperechoic cortical surface with posterior acoustic shadowing, while the joint capsule appears as a hyperechoic linear structure superficial to the condyle. The parotid gland is visible superficial and lateral to the temporomandibular joint as a heterogeneous echogenic structure, and the parotid duct (Stensen’s duct) may be identified as a tubular, hypoechoic structure within the gland. These anatomical structures serve as reference landmarks during measurement of the lateral capsule-condyle distance.

**Fig. 8:**
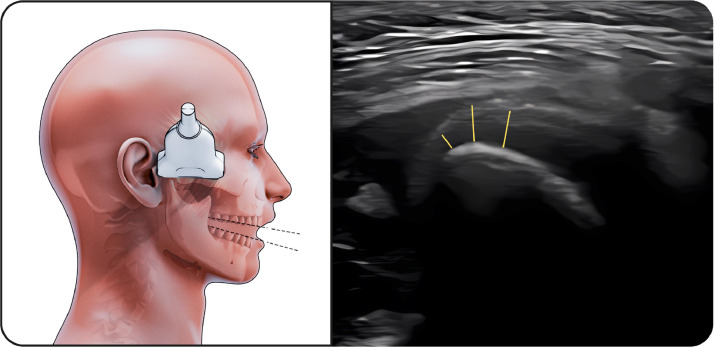
Anterior capsule-condyle distances. During ultrasonographic examination using a transverse scan, mouth opening results in anterior translation of the hyperechoic mandibular condyle, accompanied by widening of the anterior capsule-condyle distance. The cortical surface of the condyle appears as a hyperechoic line with posterior acoustic shadowing, while the joint capsule is visualized as a hyperechoic structure anterior to the condyle. The articular cartilage is identified as a thin, hypoechoic layer covering the condylar surface and is visible between the hyperechoic cortical bone and the joint space, the relative position of which changes during mouth opening as the mandibular condyle translates anteriorly. These structures allow standardized assessment of anterior joint space width and dynamic changes during mandibular movement.

Ultrasonographic examination of the TMJ included longitudinal and transverse scans in closed- and open-mouth positions to assess condylar mobility, joint space changes, and bilateral asymmetry. Three lateral capsule-condyle distances (Fig. 7) and anterior capsule-condyle distances (Fig. 8) were measured bilaterally.

## TMJ anatomical changes

TMJ evaluation included assessment of articular surface integrity, cartilage surface, articular disc morphology, and position, disc ligament integrity, and the presence of joint effusion. Each anatomical feature was scored dichotomously as present (1) or absent (0). Right and left joints were evaluated separately and compared to determine bilateral asymmetry.

The proportion of discordant bilateral findings was expressed as a percentage to derive the composite anatomical asymmetry index for each participant.

### Asymmetry indices

Muscle and interarticular space asymmetry measurements were performed by trained examiners using standardized ultrasonographic protocols. Asymmetry indices were calculated to quantify bilateral differences between the right and left sides.

Bilateral quantitative measurements were obtained in millimeters under predefined and standardized probe positions. The asymmetry index was calculated as the percentage difference between paired right- and left-side measurements using the formula: (R−L)/(R+L)×100, providing a quantitative estimate of bilateral asymmetry. This approach was consistently applied to all quantitative muscle and interarticular space measurements in the study.

### Muscle and interarticular space asymmetry

For muscle-related parameters, asymmetry indices were calculated using bilateral percentage differences derived from paired right- and left-side measurements.

Quantitative measurements were performed for all predefined masticatory muscles, including the upper, middle, and lower portions of the masseter muscle, the temporalis muscle, the lateral pterygoid muscle, and the sternocleidomastoid muscle, resulting in a total of 29 muscle-related measurements per side.

Interarticular space measurements included anterior and lateral capsule-condyle distances assessed at three predefined points on each side, resulting in 18 interarticular space measurements per side.

For each individual quantitative measurement, the absolute difference between right- and left-side values was converted into an asymmetry percentage according to the applied asymmetry index formula. The same calculation approach was applied consistently to all measurements, and each measurement contributed equally to the analysis.

For each participant, muscle asymmetry and interarticular space asymmetry indices were calculated as the arithmetic mean of the asymmetry percentages derived from the respective sets of measurements. These indices were analyzed descriptively and on an exploratory basis and were not included in the composite anatomical asymmetry index.

Measurement reliability was assessed by calculating method error using Dahlberg’s formula: SE=√((∑d²)/2n), where d represents the difference between paired measurements and n denotes the number of double measurements.

### TMJ anatomical asymmetry and composite index

Temporomandibular Joint (TMJ) anatomical alterations were assessed bilaterally and recorded as dichotomous variables, with the presence of a given feature coded as 1 and its absence as 0. A total of 11 predefined anatomical features were evaluated for each participant. Anatomical asymmetry was defined as discordance between the right and left joints for a given feature (1 vs. 0 or 0 vs. 1), whereas concordant findings (0 vs. 0 or 1 vs. 1) were considered symmetric.

A composite anatomical asymmetry index (%) was subsequently calculated at the participant level by aggregating the dichotomously assessed TMJ features. The index represents the percentage of asymmetric anatomical findings between the right and left temporomandibular joints relative to the total number of assessed features and was used exclusively for descriptive and exploratory analyses in this pilot feasibility study.

### Operational reference values

For descriptive purposes, operational reference values were applied (5% for muscle asymmetry, 15% for interarticular space asymmetry, and 25% for TMJ anatomical asymmetry). These values were not intended as diagnostic cut-offs. Different operational values were selected to reflect expected differences in biological and measurement variability across domains: muscle thickness and width measurements typically exhibit lower variability under standardized probe positioning, interarticular space measurements are more sensitive to probe angulation and mandibular position, and the TMJ anatomical asymmetry index is based on dichotomous features representing the proportion of discordant findings rather than continuous distance measures.

These operational reference values were applied to facilitate descriptive interpretation and hypothesis generation and require validation in future larger, blinded studies.

### Statistical analysis

Statistical analyses were performed to evaluate asymmetry indices and their associations with temporomandibular disorder-related clinical symptoms within the context of a pilot feasibility study. Continuous variables were summarized using descriptive statistics. To assess whether the composite anatomical asymmetry index differed from symmetry, a one-sample *t*-test was applied using zero as the reference value.

Associations between anatomical asymmetry indices and clinical symptoms were evaluated using Spearman’s rank correlation coefficient (ρ) due to the small sample size and the non-normal distribution of symptom variables.

Receiver operating characteristic (ROC) curve analyses were conducted to examine the discriminative performance of anatomical asymmetry indices for selected clinical symptoms. All statistical analyses were interpreted descriptively in view of the pilot design. A p-value < 0.05 was reported without inferential intent.

Statistical analyses were performed using standard statistical software.

## Results

All ultrasonographic measurements were recorded, and asymmetry indices were calculated for each participant. Asymmetry indices were derived from bilateral percentage differences of quantitative muscle and interarticular space measurements, as well as from dichotomously assessed TMJ anatomical features. Clinical symptoms commonly associated with Temporomandibular Disorders (TMD), including pain, joint sounds (crepitation or clicking), limited mouth opening, mandibular deviation, and tinnitus, were evaluated and documented dichotomously as present or absent (Table 1). The composite anatomical asymmetry index differed from symmetry (mean ± SD: 18.45% ± 17.13%). A one-sample *t*-test indicated that the mean value was greater than zero (*p* < 0.001); this finding is reported descriptively in view of the pilot design.

**Table 1:** Demographic characteristics and clinical symptoms of the study participants: Demographic characteristics (age, sex) and temporomandibular disorder-related clinical symptoms of the individual study participants. Clinical symptoms, including pain, joint sounds (crepitation or clicking), limited mouth opening, mandibular deviation, and tinnitus, were recorded dichotomously as present (+) or absent (-).

ID	Age (years)	Gender	Pain	Joint sounds	Limited mouth opening	Other symptoms
TDMDR1P1	24	Male	-	+	-	+
TDMDR1P2	23	Female	+	+	+	-
TDMDR1P3	22	Female	+	+	-	-
TDMDR1P5	20	Female	+	+	-	-
TDMDR1P6	26	Female	-	+	-	+
TMDR1P9	22	Male	-	+	-	-
TMDR1P10	19	Male	-	-	-	+
TMDR1P12	26	Female	-	-	-	+
TMDR1P13	22	Female	-	+	-	-
TDMDR1P4	20	Female	-	-	-	-
TDMDR1P7	23	Female	-	-	-	-
TDMDR1P8	24	Male	-	-	-	-
TDMDR1P11	24	Female	-	-	-	-
TDMDR1P14	20	Female	-	-	-	-
TDMDR1P15	25	Male	-	-	-	-

Abbreviations: +, symptom present; -, symptom absent.

Other symptoms include mandibular deviation during opening and tinnitus.

Associations between anatomical asymmetry and clinical symptoms were explored using Spearman’s rank correlation analysis. A high correlation coefficient was observed between anatomical asymmetry and joint sounds (crepitation) (Spearman’s ρ = 0.88). Pain showed a moderate positive association with anatomical asymmetry (Spearman’s ρ = 0.51). No consistent association patterns were observed between anatomical asymmetry and limited mouth opening, mandibular deviation, tinnitus, or other examined clinical symptoms.

For exploratory and descriptive purposes, suggested asymmetry reference values (5% for muscle asymmetry, 15% for interarticular space asymmetry, and 25% for anatomical asymmetry) were applied to illustrate relative differences in asymmetry severity across participants. Participants in whom multiple asymmetry indices exceeded the applied reference values more frequently reported TMD-related symptoms, whereas participants with isolated elevations in a single index more often reported no overt clinical symptoms. These observations are descriptive and intended to support hypothesis generation rather than diagnostic classification.

Table 1 summarizes participant-level demographic and clinical characteristics, while Table 2 presents asymmetry indices derived from bilateral ultrasonographic measurements of the masticatory muscles, interarticular spaces, and TMJ anatomical features. Summary statistics (mean ± SD and median [IQR]) are provided for continuous variables.

**Table 2:** Ultrasonography-derived asymmetry indices of the masticatory system: Individual ultrasonography-derived asymmetry indices of the masticatory system, including muscle asymmetry index, interarticular space asymmetry index, and Temporomandibular Joint (TMJ) anatomical asymmetry index, calculated from bilateral measurements. Asymmetry indices are expressed as percentages. Summary statistics are presented as mean ± standard deviation and median [interquartile range]. All asymmetry indices were calculated on an exploratory basis. Proposed operational reference values [5% for muscle asymmetry (+), 15% for interarticular space asymmetry (‡), and 25% for TMJ anatomical asymmetry (*)] were applied for descriptive purposes only and are not intended as diagnostic thresholds.

ID	Muscle asymmetry (%)	Interarticular space asymmetry (%)	TMJ anatomical asymmetry (%)
TDMDR1P1	9.26^a^	18.69^b^	59.09^c^
TDMDR1P2	9.41^a^	15.56^b^	27.27^c^
TDMDR1P3	8.09^a^	12.18	36.36^c^
TDMDR1P5	6.23^a^	7.88	27.27^c^
TDMDR1P6	7.38^a^	14.21	31.82^c^
TMDR1P9	4.85	16.14^b^	22.73
TMDR1P10	1.66	20.84^b^	18.18
TMDR1P12	1.34	16.28^b^	22.27
TMDR1P13	3.88	19.06^b^	22.73
TDMDR1P4	3.12	6.23	0
TDMDR1P7	2.61	14.44	0
TDMDR1P8	1.56	10.91	0
TDMDR1P11	1.36	13.65	4.55
TDMDR1P14	0.91	5.97	0
TDMDR1P15	0.88	3.15	4.55

Proposed operational reference values were applied for descriptive purposes only:

a Muscle asymmetry ≥5%.

b Interarticular space asymmetry ≥15%.

c TMJ anatomical asymmetry ≥25%.

## Discussion

Previous studies have demonstrated associations between craniofacial and temporomandibular asymmetry and Temporomandibular Disorders (TMD). Significant differences in mandibular condylar asymmetry between individuals with and without TMD symptoms have been reported using three-dimensional Computed Tomography (3D-CT),^8^ and craniofacial as well as shoulder asymmetry has been associated with TMD in larger patient cohorts.^25^^,^^26^ In addition, bilateral comparisons of masticatory muscles have suggested that muscle function may influence condylar positioning and cartilage alignment, underscoring the relevance of muscle assessment in temporomandibular disorders.^5^

Within this context, ultrasonographic evaluation of masticatory system asymmetry represents a potentially valuable area for further investigation. Ultrasonography offers a non-invasive, radiation-free, and readily accessible imaging modality that can be applied in a chairside setting with relatively short examination times. In the present pilot study, associations were observed between anatomical asymmetry indices and selected TMD-related clinical symptoms, particularly joint sounds. These findings primarily support the feasibility of ultrasound-based asymmetry assessment**,** while acknowledging the exploratory nature of the analysis.

The present study was designed to propose a structured ultrasound examination protocol and to generate preliminary data on asymmetry patterns in young adults. The asymmetry reference values applied were used solely for descriptive illustration of relative differences between participants and may serve as preliminary, operational reference points pending validation in future studies. Although the sample size was limited and the study population was relatively homogeneous, these characteristics are consistent with the pilot nature of the investigation and provide a focused framework for methodological development.

Several methodological aspects identified in this study also inform future research directions. Examiner blinding was not implemented, and the cross-sectional design precludes assessment of temporal relationships between asymmetry and symptom development. To address these points, future investigations should include larger and more diverse populations, blinded assessments, and longitudinal study designs. Such studies will be essential to determine whether ultrasonographically detected asymmetry precedes symptom onset, reflects adaptive or pathological changes over time, or is associated with symptom development.

In line with previous reports, ultrasonography has been proposed as a chairside imaging modality that may be incorporated into routine dental examinations for functional assessment of the temporomandibular joint and masticatory muscles.^11^ Within this context, the present protocol contributes to the methodological groundwork required for standardized ultrasound-based assessment of masticatory system asymmetry.

Overall, the present findings support the continued methodological development and future validation of standardized ultrasound-based protocols for assessing masticatory system asymmetry and provide a feasibility-based framework for future confirmatory studies.

Building on the present findings, the authors plan to validatethe proposed ultrasound-based asymmetry assessment in larger and more diverse cohorts, including correlation with DC/TMD diagnostic criteria. Future longitudinal studies integrating complementary imaging modalities and functional assessments are also planned to further explore the clinical relevance and temporal stability of ultrasound-detected asymmetries.

## Conclusion

This pilot study demonstrates the feasibility of a standardized ultrasound-based protocol for the quantitative assessment of masticatory system asymmetry. The findings indicate that ultrasonographic evaluation can capture measurable asymmetry patterns and exploratory associations with selected temporomandibular disorder-related symptoms, particularly joint sounds. Rather than establishing diagnostic criteria or clinical cut-off values, the present work provides a structured methodological framework and preliminary, descriptive reference values to support future research.

With further validation in larger, blinded, and longitudinal studies, ultrasound-based asymmetry assessment may contribute to improved understanding of asymmetry patterns in temporomandibular disorders. At present, the findings should be interpreted within the context of a feasibility study and are intended to support methodological development and hypothesis generation rather than clinical decision-making.

## Conflicts of interest

The authors declare no conflicts of interest.

## Data Availability

The datasets generated and/or analyzed during the current study are available from the corresponding author upon reasonable request.
